# The Shaman and Schizophrenia, Revisited

**DOI:** 10.1007/s11013-023-09840-6

**Published:** 2023-11-30

**Authors:** Tanya Marie Luhrmann, John Dulin, Vivian Dzokoto

**Affiliations:** 1 Department of Anthropology, Stanford University, Stanford, USA; 2 Department of Anthropology, Utah Valley State University, Orem, USA; 3 Department of Psychology, Virginia Commonwealth University, Richmond, USA

**Keywords:** Schizophrenia, Shamanism, Voices, Psychosis, Apprenticeship

## Abstract

This paper presents evidence that some—but not all—religious experts in a particular faith may have a schizophrenia-like psychotic process which is managed or mitigated by their religious practice, in that they are able to function effectively and are not identified by their community as ill. We conducted careful phenomenological interviews, in conjunction with a novel probe, with okomfo, priests of the traditional religion in Ghana who speak with their gods. They shared common understandings of how priests hear gods speak. Despite this, participants described quite varied personal experiences of the god’s voice. Some reported voices which were auditory and more negative; some seemed to describe trance-like states, sometimes associated with trauma and violence; some seemed to be described sleep-related events; and some seemed to be interpreting ordinary inner speech. These differences in description were supported by the way participants responded to an auditory clip made to simulate the voice-hearing experiences of psychosis and which had been translated into the local language. We suggest that for some individuals, the apprenticeship trained practice of talking with the gods, in conjunction with a non-stigmatizing identity, may shape the content and emotional tone of voices associated with a psychotic process.

## Introduction

One of the trickiest challenges in anthropology is how to interpret the relationship between cultural models of experience and what people experience phenomenologically. Anthropologists rely largely on interviews in order to understand the subjective dimension of human life: we ask people what they feel, think, hear, see, remember, and so forth. We take for granted that cultural models shape subjectivity, but also that these cultural models do not determine subjectivity. The sensitive anthropologist often needs to probe carefully and thoughtfully, because cultural models can deeply shape what people think they should experience, and people then sometimes hide feelings they believe that they should not share, or do not recognize experiences they believe that people should not have. For example, in the United States of the 1950s, the expectations that the good woman married a man who was the primary earner for the family carried with it many implicit and explicit models about sexuality, about bodies, about authority, and about social roles. These models shaped many lives and many subjective judgments, which generated both comfort and great pain. The explosive power of books like *The Feminine Mystique* (Friedan, 1963) and *The Second Sex* (de Beauvoir, 1949) arose because they allowed women to name an anger they did not always realize that they felt, and aspirations they did not always feel they could safely assert.

This complex relationship between cultural model and subjective experience is particularly complicated when we attempt to understand whether and, if so, how, culture may shape psychosis. “Psychosis” is the term we use for perceptions and thoughts which are radically askew from locally shared understandings of reality. It can be hard enough when an anthropologist is in her native social world to understand whether someone is psychotic or simply idiosyncratic. It becomes harder when that person is located within a very different set of cultural understandings. This complexity is at the heart of some of the deepest arguments about whether and how much cultural practices can change the experience of psychiatric illness, and in particular, whether and how much cultural practice can change the experience of our most troubling psychiatric illness, schizophrenia.

This article provides a blueprint for how we might answer that question. It suggests that it is plausible that a non-pathological identity and a practice which trains the way someone relates to their voices may shape the content and emotional tone of the voices associated with schizophrenia and its close clinical relatives.

## Background

One of the oldest questions in anthropology is whether “our” schizophrenia is “their” shamanism, whether someone diagnosed with schizophrenia in the modern west might become a shaman in some other society and so avoid the stigma and disability we associate with the condition today. The question emerged in the earliest days of the discipline, when the discovery of cultural difference seemed to offer freedom from blinkered presumptions about what was natural and inevitable. In 1934 Ruth Benedict published an essay, “Anthropology and the Abnormal” that became a foundational statement for this new way of thinking. Normal, she argued, was a relative concept, not an absolute one (Benedict, 1934) One of her most famous examples was madness.

It does not matter what kind of ‘abnormality’ we choose for illustration, these which depict extreme instability or those which are more in the nature of character traits, like sadism or delusions of grandeur or of persecution, there are well-described cultures in which these abnormals function at ease and with honor, and apparently without any danger or difficulty to the society. (1934: p. 60)

There were cultures, Benedict argued, where those who heard voices interpreted them as voices of spirit, and others agreed—and that could change who the person who heard voices was able to become.

In time, the claim became a war cry. Books by Laing (1960), Michel Foucault (1960), Thomas Szasz (1961), and Thomas Scheff (1966) insisted passionately, each in their own way, that psychiatry imposed meaningless standards of normalcy on artists and rebels who were simply unconventional, not actually ill. In another world, these books suggested, people diagnosed with schizophrenia would be holy men, shamans, and prophets, not treated as abnormal and as ill. There were those who protested. George Devereux, the most vehement of these voices, said: “Briefly stated, my position is that the shaman is mentally deranged” [1956 (2000): p. 226]. But he was arguing against a dominant position.

The perspective was consonant with the psychoanalysis of the day. These were the years that psychoanalysis dominated psychiatry, and to some extent anthropology, in the United States. In the 1930s, Margaret Mead, Ruth Benedict, Edward Sapir, Gregory Bateson, Ralph Linton, and others ran in psychoanalytic circles; they were part of an interdisciplinary seminar at Columbia with psychoanalysts like Abram Kardiner.^1^ From a psychoanalytic perspective, schizophrenia was a reaction to social experience—not an organic illness. Even if one conceded some organic process, there were no absolute standards against which individuals could be declared ill. To be sure, in this period “schizophrenia” was a capacious category, and many who would now be diagnosed with bipolar disorder, borderline personality disorder, or post-traumatic stress disorder were given the label (Luhrmann, 2000).

Then American psychiatry changed, in large part because many of the sickest patients did not respond to psychoanalytic therapy, and because of the availability of new pharmaceutical treatments. Psychoanalysis was replaced by a biomedical model which held that psychiatric illnesses were diseases like any others in medicine. In the new biomedical approach, schizophrenia was represented as a disease of the brain; indeed, a well-known book on schizophrenia written by a leader in the new psychiatric science was entitled *The Broken Brain* (Andreason, 1984). More narrowly defined in the new nosology, the disorder was diagnosed by a characteristic combination of thoughts (delusions) and perceptions (hallucinations) which seemed untethered to what was real and which had lasted for six months or more and caused substantial impairment in everyday life. In this new biomedical model, the characteristic symptom of schizophrenia was voices, or auditory hallucinations, which are often negative and difficult for the one who hears to handle (see Verduin et al., 2021).

Anthropology played an important role in this shift. Work in Ghana (Field, 1960) and Nigeria (Leighton et al., 1963) had already established that a pattern of experience like schizophrenia was present outside of the west. Then in 1976, the anthropologist Jane Murphy published an essay in *Science* which set out to show that whether on the Arctic or the equator, human societies identified a pattern of behavior in which some people had deluded thoughts and distorted perceptions, and other people inferred that there was something wrong with their minds. She had spent 1954-5 in a Yupik-speaking village on an island in the Bering sea, an ice-bound place where the nomadic people wore layers of fur and survived the winter by eating raw fish. They used the word *nuthkavihak*, which they translated as ‘being crazy,’ to describe people who screamed as invisible beings, or drank urine, or believed that their husbands had been murdered by witchcraft but nobody else agreed. In their villages they had shamans who behaved oddly (falling on the floor, crawling like a dog), but people did not call them *nuthkahavik*. As one person told Murphy, “when the shaman is healing [e.g. in the strange state in which he summons spirits], he is out of his mind, but he is not crazy” (1976: p. 1022)

Murphy saw the same thing among the Egba Yoruba, with whom she lived for many months in 1961 and 1963. The Yoruba, a farming people who lived in tropical thatched huts where chickens pecked in the ochre earth, used the word *were* to describe

Hearing voices and trying to get other people to see their source though none could be seen, laughing when there is nothing to laugh at, talking all the time or not talking at all, asking oneself questions and answering them, picking up sticks and leaves for no purpose except to put them in a pile, throwing away food because it is thought to contain juju, tearing off one’s clothes, setting fires, defecating in public and then mushing around in the feces, taking up a weapon and suddenly hitting someone with it, breaking things in a state of being stronger than normal, believing that an odor is continuously being emitted from one’s body. (1976: p. 1022)

In all these places, the mere fact of hearing voices was not sufficient to get tagged as ‘being crazy.’ These were worlds in which some people talked with spirits and spirits answered back. *Nuthkavahik* and *were* were used for people who seemed not to think and perceive in a way that the village considered to be normal, and what was abnormal was abnormal in the same way everywhere. For that matter, Murphy saw the same pattern in places she had lived for shorter periods, in Gambia, the Sudan, and in South Vietnam. “Symptoms of mental illness,” Murphy concluded, are “manifestations of a type of affliction shared by virtually all mankind” (1976: p. 1027). In 1980, the American Psychiatric Association released DSM III, a nosology which presented psychiatric illnesses as symptoms of medical disease.

In this new environment, it seemed foolish for anthropologists to raise the question of whether a practice like shamanism could mitigate schizophrenia, a condition now understood as a universal brain disease best treated by medication. Instead, one found in the anthropological literature papers which demonstrated that a shamanic state was not, in fact, like schizophrenia at all (Noll, 1983). Psychiatric anthropology began to focus on the way biomedical illness was shaped by local cultural conceptions. For some decades, the field focused more on disorders like depression and post-traumatic stress disorder, which seemed less obviously disease-like and where cultural influence could perhaps be more clearly understood (e.g., Kleinman, 1982; Young, 1995; Kitanaka, 2011). To be sure, even in those early years, there was important work on schizophrenia (for example, Estroff, 1981) and more work soon emerged (for example, Desjarlais, 1997; Good, 1997; Lovell, 1997; Hopper, 2003; Jenkins and Barrett, 2004; Wilce, 2004; Halliburton, 2004; Sousa, 2009; Myers, 2015; Pinto, 2014; Jenkins, 2015). Still, scholars mostly did not return to the old shamanism-and-schizophrenia concern.

It is time to take up the question of shamanism and schizophrenia, or rather religious expertise and shamanism, anew.^2^ In the last few decades, psychiatry has changed again. Many psychiatric scientists now understand psychosis, the more general term for disorders of thought and perception, to be more like a continuum than like a category, more like a trait distributed in the general population, like height, and less like a broken arm, a condition which is clearly not healthy (Peters et al., 2016, McGrath et al., 2015, van Os et al., 2009). There is sharply increased recognition that many social features play a role in who develops schizophrenia. Poverty, immigration status, trauma, and other forms of social defeat—even having a dark skin in a white society—increase the risk that someone will develop schizophrenia, a finding in line with the social disparities epidemiology in which poverty, stigma, and social defeat increase the risk for many diseases (Selten & Cantor-Grae, 2005, Luhrmann, 2007). New non-pharmacological treatments for people who hear distressing voices, a major symptom of schizophrenia, find that social practices can mitigate the destructive emotional content of what the voice says (Birchwood & Trower, 2006, Leff et al., 2014, Ruddle et al., 2011, Longden et al., 2022). The question of whether and if so how culture might affect schizophreniform disorder is now an active question in psychiatric science.

In this paper, we raise the possibility that some—but not all—religious experts have a schizophrenia-like process which is managed or mitigated by religious practice, so that individuals are able to function effectively and not identified by others in the community as ill. We suggest that the practice of the faith, and their training in apprenticeship, may diminish the negative voices so characteristic of schizophrenia and enable the individual to develop reasonable relationships with other voices that come to be experienced as positive. We suspect that the faith practice does this by offering a framework to interpret voice-hearing as intelligible within a religious framework, and a practice to help someone to work with their voices. Our evidential base for this work comes from a study of the okomfo community around Cape Coast, Ghana.

### The cultural model: the world of the okomfo

“Okomfo” is the name for the ritual specialists in the traditional religion of Ghana. (“Traditional” is a term used by practitioners and the category used in the national census of Ghana.) The practice is broadly similar to other faith practices in sub-Saharan Africa which are presumed to predate Christianity and which have spread around the world as santeria, candomblé, voodoo, bahia, and so forth. The okomfo are priests. They are understood to have special relationships with a range of greater and lesser spirits which are in some sense housed within a shrine which they tend. Typically, the okomfo develops a relationship with one god, who calls or chooses him or her. The okomfo who accepts the call is then trained by apprenticeship with more senior okomfo in the ritual procedures for interacting with the god. Over time, the okomfo may acquire relationships with other gods and spirits. Each will have their own altar in the shrine, with special objects known to be liked by the god—perhaps a mirror or a coffeecup, a doll or a ball or a bell. At intervals, the priest feeds each god according to his or her wants—the blood of sacrificed animals, perhaps annually, and schnapps, more frequently. Other people will come as clients to the shrine, asking for protection from dangerous supernatural forces, or healing and other needs. Okomfo are also trained in the use of medicinal herbs, and okomfo are sometimes the first resource when someone falls ill (for further discussion see Dulin, 2020 and de Witte, 2008).

One sees advertisements for individual okomfo along the road in different parts of Ghana. Some priests are wealthy, perhaps paid as if on retainer by local businessmen. They have large compounds where many people live. Others are poor and struggle. Each okomfo works individually, but they do gather collectively for initiations, for funerals, and for celebrations. They often wear loose caftans rather than the impeccable western attire of middle class Ghanaians. Other people sometimes refer to them as “dirty” and describe them as dealing with demonic forces. Yet most of their clients are Christians of some kind, even if they consult with them furtively and in secret.

These are people who talk with the gods. When consulted by a client, the god will enter the okomfo, who then becomes a living presence of the god for the human who has come to consult. Sometimes the possessed okomfo speaks in ways that the client will understand. Sometimes, however, the possessed okomfo’s speech will need to be interpreted for the client by a second person associated with shrine, the obrafo. After the god has gone, the okomfo will often, but not always, be amnestic for all that has happened during the possession. The god can, however, talk with the okomfo outside of possession, through dreams, visions, and words heard out loud or in the mind. Many okomfo talk easily about what a god has told them to do, and about their conversations and relationships with their gods.

The okomfo we met in pursuit of this project more or less shared a common understanding—a “cultural model”—of why and how someone would become an okomfo. The first step was that the god called the human, often out loud, often when the human was in his or her late teens—the age at which many who will develop schizophrenia have their first psychotic episode. Then at some point, the god possessed the human, who then reacted in the chaotic and sometimes violent way people do when they are newly possessed. The god often had to call more than once, because it is assumed that the person who is called will resist the call—to accept it, someone must drop out of school and enter an apprenticeship for at least one or more years, during which the apprentice has no sex, wears no shoes, and dresses in a sheet.

The purpose of the apprenticeship is to learn about the god who has called, to learn to know the gods more deeply, to listen to them more clearly, and to distinguish between the gods and demons, who will also begin to prowl around the new apprentice. Here too, there are many implicit and explicit models about how one hears and who speaks. Many okomfo describe drops placed in their eyes and ears to enable them to see and hear the gods; there is a ritual process to open the god’s mouth so that they will understand what the god is saying to them. Many describe the gods as speaking through a kind of whistling that everyone can hear, but which they are able to interpret as containing words. But gods also talk to some okomfo informally. Okomfo describe an apprenticeship process of learning to pay attention to what they hear, and to discern gods and helpful spirits from other spirits who speak. When apprentices have completed their training, there is an expectation that they will be on good terms with them. But it is also accepted that many okomfo fear them.

Initiates see their role as okomfo as a calling, one that can bring them good fortune and prosperity. However, they present it as if they did not have much of a choice: if one resists the call for too long, one can go mad. JD’s interlocutors told him of a man known around Cape Coast for a madness that arose because he resisted the call to serve the gods. He has since attempted to accept the call, but the gods no longer want him. TML met patients with schizophrenia in the psychiatric unit who, she was told, had refused their calling to be okomfo. Hence, local cultural models propose a link between madness and a call to serve the gods. They also posit that a full embrace of the spiritual practices and roles of an okomfo can prevent a debilitating psychotic break.

This then sets up the question: might okomfo have an incipient schizophrenia-like process that is managed through spiritual practice?

### The puzzle

We begin with a major assumption that schizophrenia and schizophreniform psychosis exist on a continuum (some people are much more ill than others) but that not all hallucination-like experiences are in fact expressions of schizophreniform psychosis, or indeed of psychosis at all. Psychosis is typically understood in the psychiatric literature as a condition in which someone has thoughts and/or perceptions which are radically at odds with normally accepted understandings of what is real. Many psychiatric conditions can involve some degree of psychosis, among them bipolar disorder, psychotic depression, obsessive compulsive disorder, and reactions to many drugs. Schizophrenia is a disorder which includes psychosis. The nosologist Emil Kraepelin famously distinguished it from bipolar disorder on the basis of its downward and deteriorating course (see Verduin et al., 2021). Despite hesitations about this claim, the person with bipolar disorder is presumed to cycle through periods of sometimes-psychotic mania and depression, but to return to normal functioning (“baseline”) between these periods; the person with schizophrenia is often presumed never to return to baseline functioning, even though within one person’s life, there are periods in which the psychosis is more pronounced. Schizophrenia is also associated with tangential, somewhat incoherent speech, and with impoverished facial expression, usually called flat affect. It is also often associated with striking cognitive disorganization, sometimes due to the distractions of frequent hallucinations, but often not. It is not uncommon for persons who develop schizophrenia to experience their first psychotic episode in their late teens, particularly if they are male.

The characteristic symptom of schizophrenia is hearing a voice in the absence of a speaker (see the current clinical discussions in Verduin et al., 2021). These voices are often, but not always, auditory. People with schizophrenia often report an array of voice-like-events which command, insult, and comment. They can hear voices every day, every hour, sometimes continuously, as if their heads were stuck in a beehive of harassing sound, but sometimes the voices are less frequent. There are often, but not always, negative voices. Sometimes people say that they hear sounds like rats scuttling across a field behind them. Sometimes they say that they feel the intention of a great energy, but they have to listen carefully so that they can put words to what the energy has to say. Often there are positive voices also in the mix, and neutral voices that simply comment: “She is opening the door” “She is eating lunch.” Characteristically, there are periods when someone is overwhelmed by their voices and periods when the voices seem calm. Clinical wisdom accepts that many people find that their voices diminish in intensity when they move into their forties or fifties.

In previous work, we have found that local culture appears to shape the content of voices among those diagnosed with schizophrenia (Luhrmann et al., 2015, Lebovitz et al., 2021). Study participants in San Mateo, California, reported voices that were strikingly more violent and less person-like than participants in Accra, Ghana, or Chennai, India. In Chennai, participants described more sexual content to their voices, and it was as if their voices seemed intent on sexually shaming them. They were also more likely to say that they heard family members speaking. More participants in Accra, too, heard the voices of people they knew, compared to the Americans, but what was striking there was that half the sample insisted that they heard God—and God told them not to pay attention to the demons.

In recent years, psychiatric scientists have moved to a more dimensional understanding of psychiatric illness. In the case of schizophrenia, this has been motivated by the observation that long-term outcomes are more varied than was once assumed (Harding et al., 1987); that psychotic-like symptoms are not uncommon throughout the general population (Peters et al., 2016; van Os et al., 2009; McGrath et al., 2015); and that no specific cause of the disorder has yet been found, despite decades of research (Schizophrenia Working Group, 2014). A condition like schizophrenia is understood to be distributed in the general population, with some people ill enough to come to medical attention, and others who have managed to cope effectively with their symptoms. This shift in understanding has fueled a hope that we will learn from those who have apparently psychotic experiences, but not a psychotic disorder severe enough to lead to clinical intervention, how the disorder might be mitigated. As one of these scientists suggests: “future research should focus on protective factors and determinants of well being in the context of PEs [psychotic experiences] rather than exclusively on risk factors and biomarkers of disease states” (Peters et al., 2016: p. 41).

At the same time, not all hallucination-like experiences are caused by a psychotic process. Our own work has contributed to this basic point. We have shown that there are hallucination-like events in the general population that are phenomenologically distinct, culturally salient and predicted both by a measure of absorption, which probes for an interest in immersive experience and vivid imagery, by the deliberate cultivation of inner sensory experience, and by a model of mind in which the mind world boundary is understood to be permeable (Luhrmann et al., 2013; Lifshitz et al., 2019; Luhrmann et al., 2021). Charismatic Christians, for example, whose religious world invites them into a personal relationship with God, learns to scan their thoughts for words and images they can take to be spoken to them by God. They have daydream-like prayer practices in which they seek to experience God with inner sensory vividness—sitting with God’s arm around them, listening for his voice. We have found that the more able these Christians are to become absorbed in their sensory experience, the more they pray, the more they commit to a porous model of mind, in which God can hear their thoughts and their prayers have power, the more they report hallucination-like experiences.

These hallucination-like events seem different from those reported by persons with psychosis. When charismatic evangelical Christians, witches, druids, and others like them report that a supernatural voice has an auditory quality, the events they report are typically rare (one specific event, maybe at most a handful), brief (typically four to six words, unless what they hear is on the cusp of sleep), and startling, but not distressing (Luhrmann, 2017). People report that they were driving, and they heard God say from the back seat, “I will always be with you.” They are startled beyond measure. But then they weep with joy. These voices are also phenomenologically different from the voices of psychosis. While those with psychosis often report a sense of physical oppression, as if the voice itself were a tangible thing, pushing on the body, the audible voices that Christians report are less physical. As one man said: “I was at the grocery store and, it wasn’t like *this* audible, but I felt my—God did a hiccup or something.” The voices of psychosis often feel alien, unwanted, almost assaultive: one woman described it as “a hostile take over of my mind.” The Christians experience God’s voice as other, but neither as alien nor as imposed or controlling. One man said: “It was certainly not unwelcome—it’s not a sense of taking over.” The commands feel less commanding and the otherness feels more intimate. Meanwhile, those with psychosis often reported an array of auditory and quasi-auditory experiences that could not be understood, such as whispering or murmuring, and they often reported multiple voices, often commenting or conversing with each other. Those experiences are likely less common for persons without obvious psychosis whose auditory voice experiences are rare, brief, and not distressing (but see Woods et al., 2015).

There are other routes to voice-hearing. Dissociation is a poorly understood but nonetheless quite important phenomenon in which people enter trance states and, in that condition, experience internal sensations as if they are externally real—to some extent, as if they are dreaming when awake. While some scientists argue that all voice-hearing is fundamentally dissociative (Longden et al., 2012) many agree that psychotic experiences are more alien, more uncontrollable, and less dream-like than dissociative experiences. Dissociative experiences are often described as more narratively rich and coherent (Seligman & Kirmayer, 2008). Dissociation is related to absorption (Roche & McConkey, 1990) and to trance (Spiegel & Spiegel, 2004). There is so-called positive dissociation, most famously associated with possession experiences (Bourguignon, 1976) and with trauma (Putnam, 1997; Young, 1995). Many clinicians report that people with dissociative disorder “hear voices.”

Voices can also emerge from the twilight period between sleep and awareness. As many as 37% of the general population report frequent hallucination-like events on the edge of sleep (the feeling of a presence in the room, the sense of falling into an abyss); voices are sometimes a part of those experiences, and sometimes independent (Ohayon et al., 1996). Sleep experiences have been striking under-researched in anthropology (but see work by Roger Lohman, e.g., 2019, Glaskin & Chenhall, 2013, Galinier et al., 2010, Mageo & Sheriff, 2021, and Hollan, 2013); they are important in understanding unusual sensory experiences like voices.

In sum, there are many routes to hearing voices, and within one body those routes can co-exist. Clinicians would use the distasteful term “comorbid,” but a more humanistic account would be that the experiences of any one individual may travel on many bodily pathways. There is, however, a general principle that emerges from this overview: the more auditory, negative, and frequent the voices, the more likely they to be associated with psychosis.

The question is then: when okomfo hear voices, what kind of voices do they hear and can a plausible case be made that any of them have a schizophreniform process?

## Methods

The work reported in this paper was done in 2016-7 in the Mind and Spirit project, a Templeton funded, Stanford-based comparative and interdisciplinary project under the direction of T.M. Luhrmann (PI), drawing on the expertise of anthropologists, psychologists, historians, and philosophers. That project asked whether different understandings of “mind,” broadly construed, might shape or be related to the ways that people attend to and interpret experiences they deem spiritual or supernatural. We took a mixed method, multiphase approach, combining participant observation, long form semi-structured interviews, quantitative surveys among the general population and local undergraduates, and psychological experiments with children and adults. We worked in five different countries: China, Ghana, Thailand, Vanuatu, and the US.

As part of this project, we conducted semi-structured interviews of spiritual experience with people of deep faith. In these interviews, we asked people whether they had ever heard a god or a spirit speak out loud to them, with follow up probes to helps us to discern the phenomenological quality of the experience (for example, “Did you hear it with your ears? Did you turn your head to see where it was coming from?”). We call this method of probing for details about participants’ experiences in the manner of a clinical interview “comparative phenomenology.” In Ghana, JD worked with Eunice Otoo, a carefully trained research assistant, to interview twenty okomfo in urban Cape Coast, mostly in Fante. VD interviewed twenty okomfo in the rural areas outside the city. Fully half of these forty participants said that they heard a god speak audibly only once a year or less often. Some of these participants said that they heard a god speak in a way they could hear with their ears once a week or more often.

TML then joined JD and VD in Ghana for several weeks. She reinterviewed five of the frequent voice-hearers that JD had interviewed, along with a few others within the okomfo community who described themselves as frequent voice-hearers to JD but who had not been interviewed as part of the initial sample. TML’s interview explored voice-hearing more deeply, in a manner closer to the intensive questions about phenomenology she and her colleagues had developed to talk to psychotic patients about their voices. The interview lasted at least an hour. It asked the participant to tell his or her story. Then it explored whether god’s voices were audible, when and how the participant heard them, what the voices said, whether they were ever negative and so forth. In all interviews by JD, VD and TML, we asked all participants the most salient questions asked in clinical interviews to determine psychosis (SCID, First 1980), whether they’d ever spent time in a psychiatric hospital and whether there had been a period in their lives when many people said they were crazy. TML also asked a question about trauma (“Some people have very tough periods in childhood. Some get beaten badly many times, or find that many people are very mean to them. Some people have sex against their will when they are very young. Has anything like that happened to you?”).

TML then played a 45 second clip of a track created by Pat Deegan, a psychologist who carries a diagnosis of schizophrenia, to represent the experience of hearing voices when psychotic. The words on the clip had been translated into Fante and voiced by our research team in Ghana. The voices were remixed with the Pat Deegan track by Nikki Ross-Zehdner to create a Fante equivalent. The short clip included some scratching sounds, some whispering, a seductive female voice which said, “You are the one I came for,” and a man’s voice which became more insulting and disgusted as the clip went on, and ended in some shouted commands, e.g.: “Don’t touch that!” To many people, the track sounds unpleasant and weird. TML asked the participant: “Is this what the gods sound like to you?” Two of these interviews were conducted in English, but the rest were largely in Fante and were translated in real time by the very capable research assistant who was fluent in English, Twi and Fante, the local languages, and worked with JD and VD. All interviews were then transcribed and if necessary translated.

In presenting the case studies below, we pay special attention to the life story as told by the person, and in particular to (1) whether their account of the chaos after the god first called really did suggest that the people around them thought they were crazy; (2) the phenomenology of the voice-hearing experience and how auditory, how frequent, and how negative the voices were; (3) their response to the voice-hearing track; (4) the way they responded to the SCID and trauma questions. We include at the end of each case TML’s clinical impression (5), recognizing that this judgment has many limitations.

## Case studies

*The first two of these case studies present people who do not report that they hear the voices of the gods frequently.*

### Hannah

Being called: When Hannah was a teen, she would sell goods on the road, as many poor Ghanaians do. While she was successful, she tended to give the money away. She went to some okomfo to see why she was so foolishly generous, and she was told that she had been chosen by a god. At age 15, they took her to start her training as an okomfo. She did not describe concern by others that she was “crazy.”

Voice phenomenology: Hannah said that she heard the gods speak out loud once a year. She did not hear chirping or whistling, but rather a voice like “I am talking to you.” it was a tiny voice, or sometimes it was a deep voice, but it was a normal voice with words nonetheless. She had visions more often, between once a week to every two or three months. She saw in a vision that someone had cursed her uncle, and she actually saw the person cursing her uncle. She was waking up from sleep, and she went to the uncle’s place to warn him to be careful. When she was in Ivory Coast, she saw a person who looked like her mother’s older sister, and she became afraid because she was alone and they were asking her a lot of questions. She turned around for a second, then looked back and they had disappeared. She later learned that the mother prayed that her sister’s ghost would come and look after her in Ivory Coast.

Response to the track: TML did not interview her and so Hannah did not hear the track.

Trauma/SCID: She said no to each SCID question.

### Sekyiwaa

Being called: Sekyiwaa was a 30-year-old professional okomfo. When she was a child and attending primary school, she broke another child’s pen. She bought her a new one, but the girl did not accept it, and began to bully her. The girl would follow her around harassing her, saying “I want my pen” “I want my pen.” Sekyiwaa told her father, who was an okonfo himself, and he consulted the gods. The gods responded that his daughter’s harrasser was a witch, and that Sekyiwaa had been chosen to be an okonfo. The mother opposed this because out of all of her children, Sekyiwaa loved school the most, and was poised to go far in her education. Nevertheless, she entered training. There was no story about Sekyiwaa being thought to be crazy.

Voice phenomenology: Sekyiwaa said that she does hear the gods with her ears, but that it sounded like birds chirping. Everyone could hear this, but only she would understand. She heard the gods speak audibly in this way between every two weeks and monthly. She usually saw the gods when she slept, during dreams.

Response to the track: she was not interviewed by TML and so Sekyiwaa did not hear the track.

Trauma/SCID: She said no to every SCID question.

*The case studies below are all frequent voice-hearers interviewed by JD and then TML. They are presented more or less in order of increasing evidence of a psychotic process.*

### Charles

Being called: Charles was twenty five years old during these interviews. He was neatly dressed and personable. He clearly loved being an okomfo. His mother had been a priestess, and while there were many children in the family, he was the one who helped her out, and would go be with her when she got possessed or had to visit other places. His account of how he was called involved a fairly standard account of becoming possessed in school. Sometimes in his primary school he would feel shaking, as if something in his body were really shaking him. When he reported this to his mother, she did not say anything, but later she told him that she had prayed to the gods that he would take over the shrine for her.

Around the time of these early possessions, he went to see the gods “fed” at a friend’s shrine. When he was walking home after dark, he saw the god standing by a tree, tall as a jinn, with a belt with bullets, and a gun. When Charles got close, he greeted him, but the god did not respond. So he went on, and when he looked back, he saw that the god was following him. He became frightened. He knocked on his mother’s door but she did not answer, so he jumped through the window. The god went into the shrine attached to the house, closing the door. When they went to the shrine to look for him, no one was there. Then his mother prayed for Charles and decided to send him for training. Before he left, the village prayed for him and gave him two sets of clothes.

Voice phenomenology: He said that he heard the gods daily, but he did not hear them speak in the normal way, talking back, and forth the way we speak. He said that he heard a kind of whistling that anyone could hear, but which he as a trained okomfo understood differently from other people. He was trained to understand the words within the sounds. He said that his mother taught him how to protect himself from evil, but he did not talk about hearing demons.

Response to the track: Charles said that the gods did not sound like the track. He did not recognize the shushing voice. He said that he never heard any spirit say something like “You are disgusting.” He never heard a god speak like the woman and it seemed that he did not hear anything like the commands. He did say that the gods commented on him, but it seemed more in the context of there being rules he needed to follow as an okomfo. “As an okomfo, I need to not fornicate, I need to not do things that are inappropriate; that is what they say.”

Trauma/SCID: He denied the SCID questions, said he had never been in the hospital because someone said he was mad, and talked about his father beating him when we asked.

Clinical impression: Charles seemed to TML to be someone who loved to do this work, loved to be in trance, and provided the accounts of trouble at school because they were part of the cultural model of being called by the gods.

### Sheila

Being called: Sheila was 22 at the time of the interview. She had first encountered the god who possessed her when she was 12. She loved school; she said that her teachers called her a brilliant student. But one morning, when she was sweeping out the classroom, she felt someone touch her back. When she turned around, she saw a man she did not know. He spoke to her, and said he wanted her to be his friend, and then someone called her and when she looked for the man again, he was gone. Now she began to have trouble in school. She would no longer remember what she was reading, and she seemed to blank out and then come to herself, sometimes after a fight. She began to see the man’s face again, against walls, and once or twice she would hear him, although mostly he communicated through sign language. Her mother was worried. She was sent to a church, to see a pastor and while she was in the church, spirits beckoned to her from under the trees outside, and she went to be with them. The pastor thought she ought to train to work with the spirits. She wanted to go back to school and so the master agreed to go have her train only for one year. But then he died, and no one remembered, and she stayed in training for three years and she never went back to school.

Voice phenomenology: Sheila began to hear the god speak frequently once she went through the ritual which opens his voice. Other people, she said, would hear birdsong, chirping, but she heard people. She did not say that she heard meaning in the chirping, but that she heard the gods “as the way we are talking now.” She said that she saw her gods every day. She said that she saw a woman with a child and others around her, and she saw a male god. She knew him, because she had already seen the face of her god. Sometimes she saw other gods as well. In the interview, it was sometimes unclear when she was referring to actual people who were presenting themselves as gods, or to spirits who could not be seen by others. But she seemed clearly to have experiences of gods who were disembodied. When asked whether she heard the gods speak in her mind or with her ears, she said: “If I am walking, I hear it in my mind, but if I am sitting down, like what I said, if something happens in my family, I feel its presence around me, talking to me. So I hear it the way we are talking now.” She heard the gods speak to her in her mind, she said, perhaps twice a week. She heard them speak out loud about three times a week. For example, she was doing a job for a client and the god wanted her to talk to the client’s father about his daughter. The god said: “Go to his workplace.”

She talked about having everyday conversations with her god, about the weather, about ordinary things. When the gods weren’t teaching her things about other people, they would just sit and chat with her. She would also go to talk with them if she had done a ritual, and the intervention had not worked. She would go talk with her gods to see what had happened, and what next to do. When she was talking to them outside the shrine, she would pray in her mind and they would speak in her mind. Inside the shrine, she would talk out loud and the god would also talk out loud. None of these conversations sounded negative, and she did not talk about hearing witches or demons.

Response to the track: When she listened to the track, she broke into laughter. Yes, the gods speak like that but not quite in that way, she said. Explaining her laughter, she said that the woman’s voice was the kind of thing she heard. But sometimes a witch will talk nicely like that as well, she commented. You need to be sharp to pick out who is really speaking, she said. Sometimes, she said, witches do really sound like the mean commanding man and sometimes the gods will sound like that too, giving instructions. And the shushing sounds: the gods often sounded like that. You need to interpret what they say, she said.

Trauma/SCID: She denied the SCID questions, although she said that once a week, she heard the gods talking to each other. She denied trauma.

Clinical impression: TML thought that she did not come across as someone with psychosis.

### Emelia

Being called: Emelia became an okomfo at 47. We attended her final initiation ceremony, an all-night affair with food and dancing and drumming, in which she danced in the flames of a fire to prove her power. She said that her grandmother had been an okomfu, and so the practice was familiar in her family, but initially she had not wanted to do it herself. She said she’d been periodically possessed for thirty years, but she specifically denied that during this period, people had thought there was something psychiatrically amiss. (“Sometimes when people are possessed, other people say they are crazy. Did people say that to you?” “They didn’t say that.”) She did however say that things were going wrong in her business—that she would sell things and leave the money under her mattress, and the next day it would be gone. And she said, as people do, that the god told her that if she did not go into training, he would destroy her.

Voice phenomenology: Before she was initiated, she said, the god would come and sit on her bed. “He will just sit by me and not say anything. He can sit there til the cock crows at dawn and then he leaves.” These days she saw her gods everyday, although it seemed that she mostly saw them in dreams or in the twilight state between sleep and awareness. She liked her main god. She said that before she was initiated, she would hear from him twice a week, in a way she could hear with her ears. The hearing had begun only recently, it seemed, and seemed to be connected to her choice to become initiated. Later she said that the gods came to talk to her at dawn. She never heard witches, or what might be glossed as negative spirits, although she said that she could see them (in Ghana, witches are humans, so this does not necessarily imply a hallucination. The interpreter said: “When she sees someone who would be a witch, she would know.”) And now, she explained, the god lives inside of her. You cannot be afraid, she said of someone that lives inside of you.

Response to track: When she heard the audio track, she said that she did hear the gods like that. In fact, she smiled, as if she really liked the track. “Some of them tell you that stop, you are disgusting. Maybe you have been immoral with someone’s husband so the god can tell you to stop doing that because you are bringing filth to him.” She heard the female voice, and the whispering sounds. Sometimes they spoke harshly, but mostly they spoke in a way that she liked.

Trauma/SCID: She denied trauma; she said she’d never been hospitalized; and she said no to the SCID questions.

Clinical impression: In the interview, TML had the sense that these gods were more like imaginary friends for Emelia, and the comment that she always saw them when she hear them made the experiences sound more like vivid daydreams than like psychosis. Yet the fact that she said that her gods sounded like the track was striking, and as we spoke to her about that track, her account of the voices sounded more like the experiences reported in psychosis.

### Okomfu Eric

Being called: Eric said that he had been chosen to be an okomfo when he was still in school, around the age of 16. “I was sent to the hospitals and different prayer camps but they could not really pin point what was wrong with me.” In one version of the story, he did go to the psychiatric hospital but they couldn’t find anything wrong; in another, he didn’t go, but the description of other people’s concern is consistent. He said that the head of his family thought that he was “going crazy.” Then a pastor had a vision of him as a traditional priest, and his parents sent him for training.

Voice phenomenology: He seemed to hear voices a lot and at least some of what he heard was negative. At first, he said sometimes he heard the voice in his mind, sometimes with his ears, sometimes in a dream. Then he insisted that what he heard was auditory, but in his ear, and soft—unless they are witches, when they are much louder. “The ghosts sort of speak in their nose or they nasalize when talking, when the god speaks, his voice is a little low but not too low. But the voice of the witches is loud.” Many of the specific stories he told about these voices turned out to be in a dream. Yet more than other people, he spoke about the loudness of the witches. “When they get meat, they talk; then it means that they are happy. And their talking is loud.” He said that he heard witches twice a week, and that he heard the gods four times a day.

He was very clear that training was important in helping the okomfo to hear good spirits and to deal with bad spirits. He said that in training, they teach you how to understand the gods when they speak, and to know when the god is coming. They are told how to deal with witches. Witches are, he said, the lowest in the spiritual hierarchy, but they are troublesome.

Response to the track: he said that it was not like his experience. He did not hear the whistling.

Trauma/SCID: He denied all SCID questions and also trauma.

Clinical impression: TML thought that compared to other participants, this participant emphasized the loudness of the voices, and the presence of negative voices. At the same time, the centrality of sleep and dreams in the narrative suggested that these experiences could be sleep events. JD reflected that whenever he went to visit Okomfo Eric, he seemed to be sleeping.

### Rebeccca

Being called: Rebecca was thirty at the time of the interview. She was in training and apprenticed to Charles. She wore only one red cloth, and no shoes. She had a very emotional face, and she was very engaging, very charming. She initially did not believe in the spirits, she said, and then it happened to her. She became an okomfu because she was living with her husband, and she used to quarrel badly—even using fists—and one day she just packed her bags and left. She started to fight with her fists at the age of 14, and had children by four different men. She seems to have smoked cannabis heavily (a risk factor for psychosis). People sometimes said that she was going mad, and that she should go to a psychiatric hospital. But people who knew the gods knew that she was married to a spirit who didn’t want her to be with anyone else, and that the spirit fought using her body. After Rebecca quarreled badly with her husband, she went back to her own family, and then the gods possessed her and she realized that she had been called.

Voice phenomenology: When we met, Rebecca had not yet been given the drops in her eyes and ears which would have helped her to hear and see the gods. She did not really hear them, she said, but she felt them communicate. Her main god would move his hands, and she could felt that she could see him do that. She said that he came twice a week—but sometimes he would stay for two days. In fact she had seen him with her eyes before she became an apprentice. She used to see a very tall man, well dressed, with chains (a jewelry item). The spirit talked to her, but she did not hear, She did not understand. So he used sign language to communicate. He used his hands, as if he were talking to a deaf and dumb person. Sometimes she saw witches, too, but mostly she felt as if they attacked her. “Sometimes I realize that I have fought to an extent that I can’t even wake up from bed. I will have bodily pains all over.” Despite saying that the gods do not yet speak to her, she said that sometimes, she said, she would hear a human talking to her (not a god)—she would hear and turn her head, but no one would be there. This happened often. She did not understand what they said. She also said that she heard the gods commenting on her about once a month. “Is that not Rebecca going somewhere?” And she heard commands: “won’t you stop” “stop what you are doing.”

Response to the track: When she listened to the track, she said: “this is what witches sound like.” She said that she heard them in dreams, although as she talked it seemed as if she heard them when awake as well. The voice of the god, she said, is like the shushing. And the gods said to her what the woman said—“I have come for you”—when they possess her. Like most of the okomfo, she was not startled by the track (other people often were). She heard the witches sometimes in the night, or dogs barking, but the gods, she said, tell them to stop. She slept now from 6 pm to 5 am, but she got up in the middle of the night often.

Trauma/SCID: Rebecca said that sometimes she heard her thoughts out loud; sometimes people took thoughts out of her mind or put thoughts into her mind. She said yes to hearing thoughts out loud; yes to thoughts in and out; no to the other questions. Asked whether she was beaten badly as a child, she said yes.

Clinical impression: She described experience considerable violence and she enacted considerable violence. TML thought that her presentation was not incompatible with psychosis, but that she might have been someone whose voices owed much to trauma.

### Stephen

Being called: Stephen was 31 at the time of the interview. Before he became an okomfo, he smoked a lot of cannabis and likely drank freely. He got into a lot of fights—he would hear a voice that would set off his violence, and after the violence he would calm down, but by then the police were involved. He was clear that the voices induced the violence. “I see it with my eyes and hear a voice saying I should stab the person.” Before he became an okomfo, people said that he was crazy, that “there was something wrong with my mind.” Once he got lost for three days, following what he now knows are the dwarves into the forest, he said. He could not find his way out. He thought he’d been gone just for an afternoon when he emerged. He talked about hearing voices and seeing things before his training.

Voice phenomenology: He said that when he went for training, he began to understand these voices and other odd sounds--chirping or the sound of boiling water—as the voices of gods, and he stopped being violent. He began to hear the gods say, this will happen, do this, do that. He said that his master made him bathe in a special bath of herbs in a cemetery at midnight, and that the witches and ghosts would come and he saw them. His master told him that he must not notice them. He did that, he said, and then he felt no more fear. The voices appear to have stopped inciting violence after that point. At the time of the interview, he had nine gods, which he seemed to hear daily. They are, he said, like his family. He also heard witches daily. “As for the witches, I hear their voices a lot.”

As the interview continued, he began to backtrack on the auditoriness of these voices. It sounded more as if he interpreted the meaning of signals in the world—also a common feature of psychosis (Jones & Luhrmann, 2016). He said it was like listening to water: when the water in a kettle is beginning to boil, it says, I am beginning to boil; then it says, I am boiling; then it says, take me off. The research assistant did not think that this was a metaphor for him. He said that when he was with his wife and the gods wanted to talk to him and it would be disruptive to talk directly, they signaled to him by turning the lights on and off in that way. He tried to explain his experience by saying, when someone is wearing slippers, you can hear in the sound of the slapping of the slippers what someone is trying to say. You can hear meaning in the sound of the slippers as the back heal slaps against the ground.

Response to the track: He said the track did not sound like his experience, and yet he asked to listen to it again, and when done he was clearly disturbed and he had tears in his eyes. It was the kind of sound, he said, that made him feel that he was going to become possessed. “If a god is coming, that is the sound that he makes.”

Trauma/SCID: He said that the voices commented on him every day: “Stephen is walking down the street.” Before training, he said, sometimes it felt that someone had placed thoughts into him mind, but now that had stopped. He said that he heard the gods talking to each other, and that once, just before they took him to the psychiatric hospital, he felt that thoughts had been taken out of his mind. He denied trauma. He also was clear that these experiences were not associated primarily with sleep, although sometimes they were.

Clinical impression: This participant left TML with the impression that he could well have a psychotic process. TML and the research assistant thought there was something odd in his presentation. His experiences were unusual, and unusually frequent, and his interpersonal style felt akin to someone with psychosis, at least in this interview.

### Ekow

Being called: Ekow was in his late thirties when we met. He had become an okomo in the footsteps of his grandfather. When he was around 14, 15, 16, he said that his grandfather became ill, and wanted him to take over her shrine. At that time, he began to hear the gods whispering in his ear. “I just started hearing some voices speaking into my ears, “do this.”” He looked over his shoulder to see who was talking—an indication that the voices were audible—but he could not see anyone. At times he saw them visually. “I just saw these three people and I saw them start like they are dancing on the sand. They are short, short like this.” He had strange dreams. His grandfather began to train him, he said, but then his grandfather died, and Ekow began to act strangely and get into fights. His aunt, or perhaps his mother, thought they should take him to a psychiatric hospital but his mother chose to take him for formal training. “My mother kept on telling me at first always they liked to take me to the psychiatrist because they thought I was getting crazy.” He said that he acted oddly for around six months.

Voice phenomenology: he was very clear that he had audible experiences, perhaps three to five days in a week. When he was young, he heard them maybe five or six times a day. Now, there one spirit in particular who worked with him, and spoke to him in a gruff voice. But he had relationships with other spirits as well. Some could possess him, but not all. “I work with different types of spirits now. But there are certain spirits that possess me, and other spirits I just work with, they cannot possess.” They spoke in different ways. Often they spoke softly, with a whisper. Often, they commanded. At one point, he fired a gun because a spirit told him to do so, and a young girl was hurt. Sometimes spirits spoke audibly, sometimes they spoke silently in his mind. Sometimes they spoke in the chirping sounds that the obrafo interpreted. Sometimes they spoke loudly but only he could hear. They did not seem to comment on his behavior. He thought that his gods were like family. But he also heard bad spirits, which were very strong and very strange, he said. His training, he said, taught him to ignore those witches.

Response to the track: Ekow recognized the track as being like his experiences. Sometimes, he said, he heard many different spirits at once. “That is the reason why you have to go under training. If you are trained, you could easily control these two voices, and you could easily know which one is saying the right thing.”

Trauma/SCID: He mostly said no to the SCID questions. He did say he’d been beaten up when he was young. “I might say that I was emotionally beaten up. Most of the people with this kind of gift, most of us passed through difficulties before we come across this kind of gift. ... Someone who is from a rich family or who is brought up from a nice home cannot easily get this kind of gift.”

Clinical impression: Ekow did not convey any sense of oddness or psychotic like qualities. He was extremely effective within the community. Yet much of what he said would raise clinical suspicions of psychosis. That presentation would be most compatible, perhaps, with a bipolar psychosis, but in any event, a psychosis process seemed plausible.

### Selina

Being called: Selina was 73 years old when we met, and the most educated traditionalist we met in Cape Coast. She worked as a secretary for the Africania Mission Church (the recent attempt to provide an institutional structure for traditionalist religion). She also worked as a traditional birth attendant and had a nurse’s degree at the training college. She was one of the busier okomfos, with 15-22 clients a week, and she was prosperous: her home was brimming with goats, dogs, and kin. She told one version of her call about being possessed in church while playing the organ. They took her to the hospital and could not revive her, so sent her for training. But there was a longer version of that story, which was that that her business crashed in her forties. She then ran out into the bush following the call of the gods and was lost for three days. When she was found, she was taken to a psychiatric hospital, but, she said, they found nothing wrong. “At that time I am not in normal properly. I had some thoughts, but they were not normal.” Unlike any other okomfo, she insisted that she was not trained by an apprentice, but by the gods themselves.

Voice phenomenology: Initially Selina talked about gods as actual people. People showed up every day to talk, and she only knew that they were spirits when they vanished, or when she realized that she could not see their feet, or after they left, and she felt dizzy. Then it became clear that they also spoke when she could not see them. “Sometimes if I go to the bush I hear that they are talking with me. They are telling me the herbs that I have to collect. But I never see them.” They woke her up out of sleep. During the day she heard their call but did not know who was speaking. She heard them stomping. Sometimes she could see them, but she knew that no one else could. Sometimes she said they came daily, sometimes she said every few days. Sometimes they sounded like snakes; you had to be careful in the bush, she said, because sometimes there really are snakes hissing. She also heard demons, every day. They seemed to give commands. There were a lot of gods as well. Sometimes they spoke softly, sometimes loudly. She heard them in dreams, and she heard them during the day. “Those who have spoken to me, they are plenty. They are many, many, many.” Most of her training took place in dreams or on the edge of sleep. “The first time, one day I was just sleeping in my house, then someone came and woke me up and sat behind me, on the chair. Then he started eating and then I started eating and then he started a conversation, conversation. He is just teaching something. something.” They taught her how to deal with the demons, how to ignore them. Now the gods spoke in many different kinds of voices. She heard them talking with each other. She heard them commented on her throughout the day. Sometimes she heard her disembodied neighbors talk to her.

Response to the track: She said that the gods sounded like the track. She thought it sounded as if the god is coming, and then as if a bad spirit was trying to stop the god from coming. (At one point in the track, a man shouts “stop it.”) She heard evil spirits like that, she said, but her gods were calm, like the woman on the track. She also heard the gods whisper, like the shushing on the track.

SCID/Trauma. Selina denied trauma, but she said that other people could sometimes hear what she was thinking; that people try to take thoughts out of her mind; that they try to put thoughts in her mind (here she seemed to be referring to negative voices: “if some dirty voices come in on me,”   by which she meant,“the devil.” In this part of the conversation, she said that the devils used to speak more often when she was younger). She said that sometimes the radio played specifically for her. In an earlier interview with JD, she had denied most of those questions.

Clinical impression: TML, JD, and the research assistant—and later, VD—agreed that her speech was not always on target and she did not always follow or answer all questions. TML thought that she might have been hearing voices during their interview. Her eyes would dart sideways as if listening to someone in the corner. TML was most confident that this participant, of all that we interviewed, might well have some kind of schizophreniform process.

### Observations

The goal of this exercise has not been to diagnose specific individuals, but to explore the possibility that some okomfo may have a psychotic or schizophreniform process and that okomfo practice may mitigate the severity of the illness. To be clear, there is no objective marker of psychosis. Moreover, none of these participants would meet formal criteria for psychosis, for those criteria require substantial dysfunction in life or in work, and all these participants are recognized as effective within their chosen profession. Nevertheless, we hope that this close phenomenological examination enables us to suggest that it is plausible that some individuals who serve as okomfo may experience a schizophreniform process. We observe that despite a common cultural model that presents the god’s call as inaugurating a period in which other people think that the okomfo-to-be is mad, not all incipient okomfo convey that other people were genuinely worried about them. We suggest that the more they do, the more it seems plausible that they experience psychosis. We observe that while all okomfo say that they speak with the gods, not all of them report that gods and spirits speak audibly, meanly, and frequently. We suggest that the more that they do, the more it seems plausible that they experience a psychotic process. We observe that many okomfo deny the standard SCID questions, and we suggest that the more someone answers in a way that seems clinically relevant, the more it seems plausible that they experience of psychotic process. And in response to our novel probe, a track made to simulate the experience of hearing voices in schizophrenia, we observe that not everyone agreed that the gods sounded like the track. When someone does, we suggest that this increases the plausibility that they experience a psychotic process.

Among our eight participants who described frequent voice-hearing, there was at least one person who clearly stated that other people thought they were crazy when she was called by the gods; who clearly experienced many audible, mean, and frequent voices even now; who said yes to many of the SCID questions; and who thought that the track sounded the way her gods sounded. This suggests that there is a wide array of phenomenological experience among the okomfo who hear the voices of the gods, and that at least one (but likely not all of them) experience a psychotic-like process and yet they are not ill.

It is always possible that those okomfo who seem to experience a psychotic-like process but are not ill were simply never very ill, and that their participation in okomfo practice has not altered their voice-hearing at all. However, given our increasingly awareness of the social shaping of schizophrenia, and of the culturally-shaped nature of psychotic symptoms, we want to suggest that okomfo-style religious practice may help the voice-hearing make more sense to the one who hears, and possibly, render the voices into a kinder form.

At least two cultural forms are likely in play.

First, practice as an okomfo offers someone an identity that does not pathologize their experience. When the person falls ill, they are able to interpret not only the voices but the social disorientation of their first psychotic episode as being comprehensible and normal—within a specific context—from the very beginning of their experience. Increasingly, psychiatric scientists suggest that the very early identification of voice-hearing as a sign of madness is extremely costly for those with psychosis in the west—that this identification provides a toxic identity from which the patient never fully recovers (Seikkula, 2003; Moseley et al., 2022). The label of “okomfo” not only proves a way to avoid this toxic identity, but allows the afflicted person to re-interpret the judgements of other people in a thorough-going manner. In India, psychiatrists and family for the most part do not use the word “schizophrenia” in conversation with the patient (Pinto, 2014). Anthropologists have suggested that this may help people to understand the illness differently, and to think that things will get better, and that perhaps sometimes they do (Sousa, 2009).

This non-pathologization is relevant for the very experience of hearing voices. In the US, for example, the primary meaning of “hearing voices” is that one is mad (Myers, 2015). This was perhaps the most consistent observation in our comparison across samples of persons with schizophrenia in San Mateo, Chennai, and Accra (Luhrmann et al., 2014). While voice hearing is indeed associated with madness in Ghana (Goldstone, 2017) it is also true that there is culturally a general disinterest in the distinction between the hallucination-like experience of hearing a voice outside the head and the experience of hearing an inner voice within (Dulin, 2020). We found that our participants repeatedly seemed to blur the boundary between inner and out, as Emelia did in this interview with TML and her interpreter Eunice (this is translated from the Fante):

Emelia: When they put the drops in your eyes and in your ears, whatever the god says you would hear it every day.

Eunice: Do you hear it with your ears or in your mind?

Emelia: You hear it with your mind. If you are doing a difficult job that wouldn’t be successful, quickly they will let you know that you can’t do it.

Eunice: Do you hear it with your ears or in your mind?

Emelia: I hear with my ears.

To be clear, Emelia, like our other participants, was able to distinguish phenomenologically between inner and outer experience after careful probing. But the general disinterest in the distinction might diminish the toxic attribution of hearing voices reported by persons with schizophrenia in the west (Luhrmann, 2016). Many years ago, the Forteses (Dolores was a psychoanalyst, Meyer an anthropologist) observed that the content of voices was more gentle in Ghana than in the UK, an observation we found as well (Fortes & Mayer, 1966). That too might make a difference to outcome.

Second, practice may alter the content and emotional tone of the voices. It is clear that participants expect that apprenticeship will change their experience of the gods. In fact, of course, training is meant to make the experience of hearing god’s voice possible. Senior okomfo place drops in the eyes and ears of novices in order to enable them to see and hear the gods. Yet as we have seen, at least some novices both see and hear the god before they get the drops. Participants are clear that training involves learning to distinguish between good spirits and bad spirits; learning to ignore bad spirits; and learning to get to know the good god who has chosen them. As Ekow said: “When you hear the voice, you have to know that it is this spirit which is speaking to you.” He was also quite clear that this training gave him a greater sense of mastery over these voices.

Okay, right now, my experience has changed a little bit. At first, when I was training, I could hear the voice but I could not control the voice. I can now control the voice. But when I finished training, I could easily hear the voice, whenever I heard the voice, I could know that, “This voice is a good voice,” or, “The direction they are giving me is the preferred direction, so I should follow it.” Because I have been trained.

What Ekow said was consistent with remarks made by other participants. People described learning more about the god and about getting to know the god, and they spoke about learning to manage witches and evil spirits, in part by learning how not to fear them and how not to respond.

There is evidence that social practices may alter the experience of hearing distressing voices. There are new, still relatively unusual and seemingly radical approaches to voice-hearing among persons with schizophrenia, mostly based in Europe (most famously, Hearing Voices groups; Longden et al., 2022). These approaches teach people not to fear their voices, and to develop a relationship with their dominant voice—in effect, to place the voice within a social relationship with the person who hears, with the expectation that the voice will the behave as if it is indeed within a social relationship and be more responsive and more engaged with the human who hears. These training practices also presume that repeated practice—not an on-and-off training, but a repeated, iterative, engagement—will make a difference to the ease with which individuals are able to feel an increased sense of control over their voices.

## Conclusion

Some decades ago there was a famous anthropological dispute about insight and cure. In *Medusa’s Hair*, (1981) Gananath Obeyesekere, who was psychoanalytically oriented, described Sinhalese priestesses who had abandoned their human husbands in order to be possessed by a god. As a mark of his acceptance of their devotion, their god gave them matted locks that sometimes rose above their heads like a cobra. The possessions were in many ways ecstatic, but the locks were itchy and their new roles as priestess forced them to give up many comforts. Obeyesekere thought that the women were drawn to this god and the matted locks he bestowed because they had intense unconscious conflicts around their sexuality, and their practice helped them to recognize and negotiate those conflicts through cultural symbols. He called this the “work of culture” (see also Obeyesekere, 1990). Melford Spiro, who was also psychoanalytically oriented, vehemently disagreed. He thought that these women were psychotic and “beyond repair, except perhaps by means of prolonged psychiatric intervention” (1997: pp. 124–5). At the heart of the dispute lay the question of whether someone must be able to name their emotional conflict explicitly before true cure is possible. To Spiro, these women needed insight—not images.

In a brilliant article, Bambi Chapin (2008), who also worked in Sri Lanka, argued that these women were not so much conflicted as traumatized (and indeed there was abundant evidence for their trauma). She pointed out that psychiatric science understood that people who had been traumatized often dissociated uncontrollably, and that the psychotherapeutic treatment of trauma often involved repeatedly putting patients into trance to in effect teach them how to control their dissociative episodes. She pointed out that observers often disagreed about the truth of the memories these patients presented (Hacking, 1995; Young, 1995) but agreed that the management of the dissociative state was crucial—and that control of the state helped patients to come to a new understanding of their lives (as in the DSM-IV-TR, American Psychiatric Association, 2000). Chapin thought that neither the western patient nor the Sinhalese priestess had insight, but both repeatedly practiced going in and out of trance in a way that helped them to manage their trauma and led them to more positive life experiences. In this telling, both western psychotherapy and Sinhalese spirit possession were examples of the work of culture. Chapin’s article was a powerful endorsement of the importance of culture practice over clinical insight.

We think what we are seeing in this story of the shaman and schizophrenia is the work of culture. Schizophrenia is a continuum. Some are better able to function, some less able, and the severity of the illness waxes and wanes across the decades. People with schizophrenia have other capabilities as well, also likely unevenly distributed across individuals—among them the capacity to imagine and the capacity to go into trance. In social worlds where there is an identity in which hearing voices is normal, and practices which teach people how to distance themselves from negative voices and develop relationships with positive voices, the work of culture may allow some people with schizophrenia-like experiences to experience less severe illness, and function effectively in the world. The work of culture includes representation, but above all it involves practice: repeatedly talking to those voices in ways that insist that the voice is a social person in a social relationship who has a social responsibility to the human who hears.

Benedict’s deepest insight was that subjective experience was “pathoplastic”: that symptoms would acquire specific form and content as they were shaped by events in a person’s life (Gold & Gold, 2012). Culture, she thought, did not have the power to completely alter subjective experience. To use another of her famous examples, someone who was sexually attracted to someone of the same sex would not necessarily find that the pattern of their sexual attraction was altered by their cultural location. But the local culture could profoundly alter the way someone felt about their orientation, and that could change everything for them. Psychiatric science has been relatively open to the idea that culture shapes the content of psychotic symptoms: in a medieval world, someone with schizophrenia could hear demons and angels, while in a modern one, they might hear robots (Kroll & Bachrach, 1982). The radical core of Benedict’s insight is that these differences in content might affect emotional meaning so deeply that the person might not even experience themselves as ill. In a deeply biomedical era which has tended to assume that schizophrenia can only be treated by medication, this could be a very important lesson for us to re-learn.

## Footnotes

1 This story is well told in John Gilkeson *Anthropologists and the Rediscovery of America, 1886–1965*; George Stocking *Malinowski, Rivers, Benedict and Others: Essays on Culture and Personality;* Robert Levine,* Culture, Behavior and Personality.*

2 We recognize that many have questioned the broad flexible definition of shamanism anthropologists have historically used in their study of religious experts (Grant, 2021). Here, we stick with the tacit consensus on shamanism that Grant identified in anthropological treatments of the subject: “To be a shaman, as opposed to an oracle, sorcerer, diviner, clairvoyant, geomancer, witch, or warlock, you should be able to engage in two-way communication or, for that matter, simply merge with spirits at will” (Grant, 2021: p. 10). This is the definition adopted in the debates we are revisiting, so we find it useful to adopt it for our current intervention.

## References

[CR1] American Psychiatric Association. (2000). *Diagnostic and statistical manual IV-TR*. American Psychiatric Association Press.

[CR2] Andreasen, N. C. (1984). *The broken brain: The biological revolution in psychiatry*. Harper and Row.

[CR3] Benedict, R. (1934). Anthropology and the Abnormal. *Journal of General Psychology,**10*(1), 59–82. 10.1080/00221309.1934.9917714

[CR4] Birchwood, M., & Trower, P. (2006). The future of cognitive-behavioural therapy for psychosis: not a quasi-neuroleptic. *British Journal of Psychiatry,**188*, 107–108. 10.1192/bjp.bp.105.014985 10.1192/bjp.bp.105.014985 16449695

[CR5] Bourguignon, E. (1976). *Possession*. Chandler & Sharp Publishers Inc.

[CR6] Chapin, B. (2008). Transforming Possession: Josephine and the Work of Culture. *Ethos,**36*(2), 220–245. 10.1111/j.1548-1352.2008.00012.x

[CR7] de Beauvoir, S. (1949). *Le Deuxième Sexe*. Gallimard.

[CR8] Desjarlais, R. (1997). *Shelter Blues: Sanity and Selfhood among the Homeless*. University of Pennsylvania.

[CR9] Devereux, G. (2000). Normal and abnormal. In R. Littlewood & S. Dein (Eds.), *Cultural psychiatry and medical anthropology. *Athlone.

[CR10] Dulin, J. (2020). Charismatic Christianity’s Hard Cultural Forms and the Local Patterning of Divine Voices in Ghana. *American Anthropologist,**123*(1), 108–119. 10.1111/aman.13523

[CR11] Estroff, S. E. (1981). *Making it crazy: An Ethnography of Psychiatric Clients in an American Community*. University of California.

[CR12] Field, M. J. (1960). *Search for security: An Ethno-psychiatric study of Rural Ghana*. Northwestern University Press.

[CR13] Fortes, M., & Mayer, D. (1966). Psychosis and Social Change among the Tallensi of Northern Ghana. *Cahiers D’etudes Africaine.,**6*(21), 5–40. 10.3406/cea.1966.3056

[CR14] Foucault, M. (1960). *Folie and Déraison [Madness and Civilization]*. Tavistock.

[CR15] Friedan, B. (1963). *The feminine mystique*. Norton. 10.2105/ajph.100.9.1582 PMC2920960 20705962

[CR16] Galinier, J., Aurore, M. B., Guy, B., Laurent, F., Francine, F., Juliette, R. P., Piero, S., Philippe, S., Michèle, T., & Iole, Z. (2010). Anthropology of the night: Cross-disciplinary investigations. *Current Anthropology,**51*(6), 819–847. 10.1086/653691

[CR17] Glaskin, K., & Chenhall, R. (Eds.). (2013). *Sleep around the world*. Palgrave Macmillan.

[CR18] Gold, J., & God, I. (2012). The ‘Truman Show’ Delusion: Psychosis in the Global Village. *Cognitive Neuropsychiatry,**17*(6), 455–472. 22640240 10.1080/13546805.2012.666113

[CR19] Goldstone, B. (2017). A prayer’s chance. *Harper’s,**5*, 41–53.

[CR20] Good, B. (1997). Studying Mental Illness in Context: Local, Global, or Universal. *Ethos,**25*(2), 230–248. 10.1525/eth.1997.25.2.230

[CR21] Grant, B. (2021). Slippage: An anthropology of shamanism. *Annual Review of Anthropology.,**50*, 9–22. 10.1146/annurev-anthro-101819-110350

[CR22] Hacking, I. (1995). *Rewriting the Soul: Multiple Personality and the Sciences of Memory*. Princeton University Press.

[CR23] Halliburton, M. (2004). Finding a Fit: Psychiatric Pluralism in South India and Its Implications for WHO Studies of Mental Disorder. *Transcultural Psychiatry,**41*, 80–98. 15171208 10.1177/1363461504041355

[CR24] Harding, C. M., Brooks, G. W., Ashikaga, T., Strauss, J. S., & Breier, A. (1987). The Vermont longitudinal study of persons with severe mental illness, II: Long-term outcome of subjects who retrospectively met DSM-III criteria for schizophrenia. *American Journal of Psychiatry.,**144*(6), 727–735. 10.1176/ajp.144.6.727 3591992 10.1176/ajp.144.6.727

[CR25] Hollan, D. (2013). Sleeping, Dreaming and Health in rural Indonesia and the United States: a cultural and experiential approach. *Social Science and Medicine,**79*, 23–30. 22705181 10.1016/j.socscimed.2012.05.006

[CR26] Hopper, K. (2003). *Reckoning with Homelessness*. Cornell University Press.

[CR27] Jenkins, J. (2015). *Extraordinary conditions: Culture and experience in mental health*. University of California.

[CR28] Jenkins, J. D., & Barrett, R. J. (2004). *Schizophrenia, culture, and subjectivity: The edge of experience*. Cambridge University Press.

[CR29] Jones, N., & Luhrmann, T. M. (2016). Beyond the sensory: Findings from an in-depth analysis of the phenomenology of “auditory hallucinations” in schizophrenia. *Psychosis: Psychological Social and Integrative Approaches,**8*(3), 191–202. 10.1080/17522439.2015.1100670 10.1080/17522439.2015.1100670

[CR30] Kitanaka, J. (2011). *Depression in Japan*. Princeton University Press.

[CR31] Kleinman, A. (1982). Neurasthenia and depression: A study of somatization and culture in China. *Culture Medicine and Psychiatry.,**6*, 117–190. 7116909 10.1007/BF00051427

[CR32] Kroll, J., & Bachrach, B. (1982). Visions and psychopathology in the middle ages. *The Journal of Nervous and Mental Disease,**170*(1), 41–49. 7033474 10.1097/00005053-198201000-00007

[CR33] Laing, R. D. (1960). *The divided self: An existential study in sanity and madness*. Penguin.

[CR34] Lebovitz, J. G., Padmavati, R., Hema Tharoor, T. M., & Luhrmann,. (2021). Sexual shaming and violent commands in schizophrenia: Cultural differences in distressing voices in India and the United States. *Schizophrenia Bulletin Open,**2*(1), sgab004. 10.1093/schizbullopen/sgab004 10.1093/schizbullopen/sgab004

[CR35] Leff, J., Williams, G., Huckvale, M., Arbuthnot, M., & Leff, A. P. (2014). Avatar therapy for persecutory auditory hallucinations: What Is It and How Does It Work? *Psychosis: Psychological Social and Integrative Approaches,**6*, 166–176. 10.1080/17522439.2013.773457 PMC4066885 24999369

[CR36] Leighton, A., Lambo, A., Hughes, C., Leighton, D., Murphy, J., & Macklin, D. (1963). *Psychiatric Disorder among the Yoruba*. Cornell University Press.

[CR37] Lifshitz, M., van Elk, M., & Luhrmann, T. M. (2019). Absorption and spirituality. *Journal of Consciousness and Cognition*. 10.1016/j.concog.2019.05.008 10.1016/j.concog.2019.05.008 31228696

[CR38] Lohmann, R. I. (2019). Culture and dreams. In D. K. Kenneth (Ed.), *Cross-cultural psychology: Contemporary themes and perspectives* (2nd ed., pp. 327–341). Wiley-Blackwell.

[CR39] Longden, E., Corstens, D., Bowe, S., Pyle, M., Emsley, R., Peters, S., Branitsky, A., Chauhan, N., Dehmahdi, N., Jones, W., Holden, N., Larkin, A., Miners, A., Murphy, E., Steele, A., & Morrison, A. P. (2022). A psychological intervention for engaging dialogically with auditory hallucinations (Talking With Voices): A single-site, randomised controlled feasibility trial. *Schizophrenia Research.,**250*, 172–179. 36423442 10.1016/j.schres.2022.11.007 PMC9754007

[CR40] Longden, E., Madill, A., & Waterman, M. G. (2011). Dissociation, trauma, and the role of lived experience: Toward a new conceptualization of voice hearing. *Psychological Bulletin,**128*, 28–76. 10.1037/a0025995 22082488

[CR41] Lovell, A. (1997). The city is my mother. *American Anthropologist,**99*, 355–368. 10.1525/aa.1997.99.2.355

[CR42] Luhrmann, T. M. (2000). *Of two minds: An anthropologist looks at American Psychiatry*. Knopf.

[CR43] Luhrmann, T. M. (2007). Social defeat and the culture of chronicity: Or, why schizophrenia does so well over there and so badly here. *Culture, Medicine, and Psychiatry,**31*, 135–172. 17534703 10.1007/s11013-007-9049-z

[CR44] Luhrmann, T. M. (2016). *I’m* Schizophrenic! How Diagnosis can change Identity in the United States. In T. M. Luhrmann & J. Marrow (Eds.), *Our most troubling madness: Case studies in schizophrenia across cultures* (pp. 27–41). University of California.

[CR45] Luhrmann, T. M. (2017). Diversity within the psychotic continuum. *Schizophrenia Bulletin,**43*(1), 27–31. 10.1093/schbul/sbw137 27872266 10.1093/schbul/sbw137 PMC5216862

[CR46] Luhrmann, T. M., Nusbaum, H., & Thisted, R. (2013). Lord, teach us to pray: prayer affects cognitive processing. *Culture and Cognition,**13*, 159–177. 10.1163/15685373-12342090

[CR47] Luhrmann, T. M., Padmavati, R., Tharoor, H., & Osei, A. (2015). Differences in Voice-Hearing Experiences of People with Psychosis in the U.S.A., India, and Ghana: Interview-Based Study. *The British Journal of Psychiatry,**206*, 41–44. 24970772 10.1192/bjp.bp.113.139048

[CR48] Luhrmann, T. M., Weisman, K., Aulino, F., Brahinsky, J. D., Dulin, J. C., Dzokoto, V. A., Legare, C. H., Lifshitz, M., Ng, E., Ross-Zehnder, N., & Smith, R. E. (2021). Sensing the presence of gods and spirits across cultures and faiths. *Proceedings of the National Academy of Sciences,**118*(5), e2016649118. 10.1073/pnas.20166491188pp 10.1073/pnas.20166491188pp PMC7865123 33495328

[CR49] Mageo, J., & Sheriff, R. E. (Eds.). (2021). *New directions in the anthropology of dreaming*. Routledge.

[CR50] McGrath, J. J., Saha, S., Al-Hamzawi, A., Alonso, J., Bromet, E. J., Bruffaerts, R., Caldas-de-Almeida, J. M., Chiu, W. T., de Jonge, P., Fayyad, J., & Florescu, S. (2015). Psychotic experiences in the general population: A cross-national analysis based on 31261 respondents from 18 countries. *JAMA Psychiatry,**672*, 97–705. 10.1001/jamapsychiatry.2015.0575 PMC5120396 26018466

[CR51] Moseley, P., Powell, A., Woods, A., Fernyhough, C., Alderson-Day, B., & Continuum, V.-H. (2022). A Phenomenology of Spiritual Voices. *Schizophrenia Bulletin,**48*(5), 1066–1074. 10.1093/schbul/sbac054 35733238 10.1093/schbul/sbac054 PMC9434432

[CR52] Murphy, J. (1976). Psychiatric labeling in cross-cultural perspective. *Science,**191*(1976), 1019–1028. 1251213 10.1126/science.1251213

[CR53] Myers, N. L. (2015). *Recovery’s Edge: An Ethnography of Mental Health Care and Moral Agency*. Vanderbilt University Press.

[CR80] Noll, R. (1983). Shamanism and schizophrenia: A state-specific approach to the “schizophrenia metaphor” of shamanic states. *American Ethnologist, 10*(3), 443–459. 10.1525/ae.1983.10.3.02a00030

[CR54] Obeyesekere, G. (1981). *1981 Medusa’s Hair: An Essay on Personal Symbols and Religious Experience*. University of Chicago Press.

[CR55] Obeyesekere, G. (1990). *The work of culture*. Chicago University Press.

[CR56] Ohayon, M., Priest, R., Caulet, M., & Guilleminault, C. (1996). Hynagogic and hynapompic hallucinations: pathological phenomena? *British Journal of Psychiatry,**169*, 459–467. 10.1192/bjp.169.4.459 8894197

[CR57] Peters, E., Ward, T., Jackson, M., Morgan, C., Charalambides, M., McGuire, P., Woodruff, P., Jacobsen, P., Chadwick, P., & Garety, P. A. (2016). Clinical, socio-demographic and psychological characteristics in individuals with persistent psychotic experiences with and without a “need for care.” *World Psychiatry,**151*, 41–52. 10.1002/wps.20301 PMC4780307 26833608

[CR58] Pinto, S. (2014). *The daughters of Parvati*. University of Pennsylvania Press.

[CR59] Putnam, F. (1997). *Dissociation in Children and Adults*. Guilford.

[CR60] Roche, S. M., & Mcconkey, K. M. (1990). Absorption: Nature, assessment, and correlates. *Journal of Personality and Social Psychology,**59*(1), 91–101. 10.1037/0022-3514.59.1.91 10.1037/0022-3514.59.1.91

[CR61] Ruddle, A., Mason, O., & Wykes, T. (2011). A review of hearing voices groups: evidence and mechanisms of change. *Clinical Psychology Review,**5*, 757–766. 10.1016/j.cpr.2011.03.010 21510914

[CR62] Scheff, T. (1966). *Being mentally Ill: A sociological theory*. Aldine Press.

[CR63] Schizophrenia Working Group of the Psychiatric Genomics Consortium. (2014). Biological insights from 108 schizophrenia-associated genetic loci. *Nature,**511*, 421–427. 10.1038/nature13595 25056061 10.1038/nature13595 PMC4112379

[CR64] Seikkula, J., Alakare, B., Aaltonen, J., Holma, J., Rasinkangas, A., & Lehtinen, V. (2003). Open dialogue approach: Treatment principles and preliminary results of a two-year follow-up on first episode schizophrenia. *Ethical Human Sciences and Services,**5*(3), 163–182.

[CR65] Seligman, R., & Kirmayer, L. J. (2008). Dissociative experience and cultural neuroscience: narrative, metaphor and mechanism. *Culture, Medicine and Psychiatry,**32*(1), 31–64. 10.1007/s11013-007-9077-8.PMID:18213511;PMCID:PMC5156567 18213511 10.1007/s11013-007-9077-8.PMID:18213511;PMCID:PMC5156567 PMC5156567

[CR66] Selten, J.-P., & Cantor-Graae, E. (2005). Social Defeat: Risk Factor for Schizophrenia? *The British Journal of Psychiatry,**187*, 101–102. 16055818 10.1192/bjp.187.2.101

[CR67] Sousa, A. (2009). Pragmatic ethics, sensible care. Ph.D. *Dissertation*. University of Chicago.

[CR68] Spiegel, D., & Spiegel, H. (2004). *Trance and treatment*. American Psychiatric Press.

[CR69] Spiro, M. E. (1997). *Gender ideology and psychological reality: An essay on cultural reproduction*. Yale University Press.

[CR70] Szasz, T. (1961). *The myth of mental illness*. Harper and Row.

[CR71] van Os, J., Linscott, R. J., Myin-Germeys, I., Delespaul, P., & Krabbendam, L. (2009). A systematic review and meta-analysis of the psychosis continuum: Evidence for a psychosis proneness-persistence-impairment model of psychotic disorder. *Psychological Medicine,**39*(2), 179–195. 18606047 10.1017/S0033291708003814

[CR72] Verdiun, M., Ruiz, P., & Boland, R. (2021). *Kaplan & Sadock’s synopsis of psychiatry*. Wolters Kluwer Health.

[CR73] Wilce, J. (2004). Madness, fear, and control in Bangladesh: Clashing bodies of power/knowledge”. *Medical Anthropology Quarterly,**18*(2004), 357–375. 15484968 10.1525/maq.2004.18.3.357

[CR75] de Witte, M. (2008). Spirit Media. PhD thesis, Univerisiy of Amsterdam.

[CR76] Woods, A., Jones, N., Alderson-Day, B., & Callard, F. (2015). Charles Fernyhough experiences of hearing voices: analysis of a novel phenomenological survey *Lancet*. *Psychiatry,**2*, 323–331. 26360085 10.1016/S2215-0366(15)00006-1 PMC4580735

[CR77] Young, A. (1995). *The harmony of illusions*. Princeton University Press.

